# A Single Lung Transplant in a Patient with Fabry Disease: Causality or Far-Fetched? A Case Report

**DOI:** 10.1155/2013/905743

**Published:** 2013-04-07

**Authors:** Martina Gaggl, Renate Kain, Peter Jaksch, Dominik Haider, Gerald Mundigler, Till Voigtländer, Raute Sunder-Plassmann, Paulus Rommer, Walter Klepetko, Gere Sunder-Plassmann

**Affiliations:** ^1^Department of Medicine III, Division of Nephrology and Dialysis, Medical University of Vienna, Währinger Gürtel 18-20, 1090 Vienna, Austria; ^2^Department of Pathology, Medical University of Vienna, Währinger Gürtel 18-20, 1090 Vienna, Austria; ^3^Department of Surgery, Division of Thoracic Surgery, Medical University of Vienna, Währinger Gürtel 18-20, 1090 Vienna, Austria; ^4^Department of Medicine II, Division of Cardiology, Medical University of Vienna, Währinger Gürtel 18-20, 1090 Vienna, Austria; ^5^Institute of Neurology, Medical University of Vienna, Währinger Gürtel 18-20, 1090 Vienna, Austria; ^6^Department of Laboratory Medicine, Laboratory for Molecular Diagnostics, Medical University of Vienna, Währinger Gürtel 18-20, 1090 Vienna, Austria; ^7^Deparment of Neurology, Medical University of Vienna, Währinger Gürtel 18-20, 1090 Vienna, Austria

## Abstract

*Introduction*. Fabry disease is a rare X-linked lysosomal storage disorder, characterized by an *α*-galactosidase A deficiency resulting in globotriaosylceramide storage within cells. Subsequently, various organ systems are involved, clinically the most important are kidneys, the heart, and the peripheral and central nervous systems. Although obstructive lung disease is a common pathological finding in Fabry disease, pulmonary involvement is a clinically disregarded feature. *Case Presentation*. We report a patient with a diagnosis of chronic obstructive pulmonary disease (COPD) who received a single lung transplant in 2007. Later, a kidney biopsy revealed the diagnosis of Fabry disease, which was confirmed by enzymatic and genetic testing. Ultrastructural changes in a native lung biopsy were consistent with the diagnosis. Although the association of a lung transplant and Fabry disease appears far-fetched on first sight, respiratory impairment cannot be denied in Fabry disease. *Conclusion*. With this case presentation, we would like to stimulate discussion about rare differential diagnoses hidden beneath widespread disease and that a correct diagnosis is the base of an optimal treatment strategy for each patient. Overall, the patient might have benefited from specific enzyme replacement therapy, especially in view of the chronic kidney disease.

## 1. Introduction

Fabry disease is a rare X-linked lysosomal storage disorder, characterized by an *α*-galactosidase A deficiency resulting in globotriaosylceramide storage within cells. Subsequently, various organ systems are involved, clinically the most important are kidneys, the heart, and the peripheral and central nervous systems. However, each patient presents with a unique pattern of organ involvement, degree of clinical symptoms, and severity of organ damage [1]. 

Although obstructive lung disease, mainly of the small airway system, is a common pathological finding in Fabry disease [2–5], pulmonary involvement is not widely appreciated by clinicians. Respiratory symptoms may be falsely interpreted as arising from cardiac dysfunction [6], and lung failure due to Fabry disease has not been reported so far.

In contrast to the respiratory system, patients with Fabry disease regularly develop organ failure predominantly involving kidneys and, less common, the heart. Renal transplantation has proven beneficial; however, recurrence of the disease in the transplant may occur [7]. Despite the fact that development of hypertrophic cardiomyopathy is considered a typical pathology and cardiac events are a leading cause of death in Fabry disease, heart failure necessitating transplantation is less frequent [6, 8]. Here, we report a patient who received a single lung transplant and later was diagnosed to suffer from Fabry disease. To the best of our knowledge, this is the first report of a patient with end-stage lung disease and Fabry disease who required a lung transplant.

## 2. Case Presentation

In 2006, a 58-year-old female retired salesperson with a seven-year history of respiratory impairment due to clinical symptoms of chronic obstructive pulmonary disease (COPD) and a continuous oxygen therapy dependency for one year was referred to the Department of Surgery at the Medical University of Vienna. The former smoker (40 pack years) was evaluated for lung transplantation and received a single lung transplant (left lung) 12 months later in 2007. 

**Figure 1:**
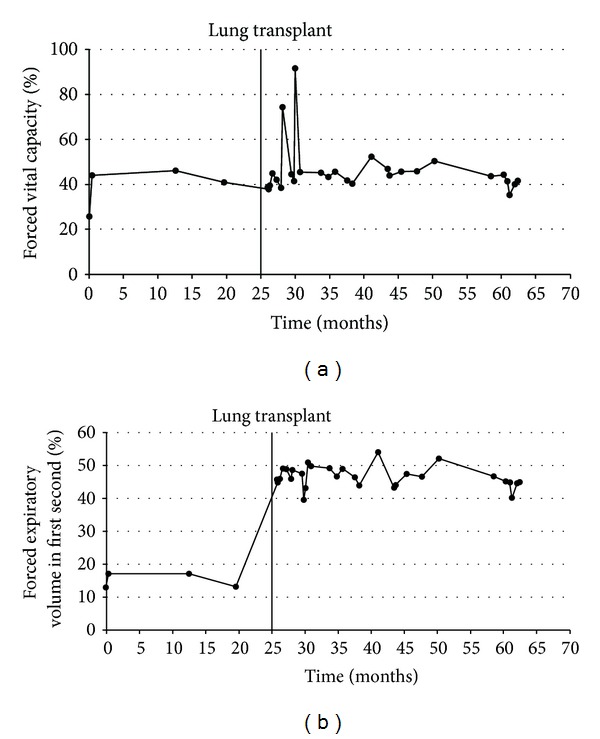
Time course of the pulmonary function parameters: (a) forced vital capacity and (b) forced expiratory volume in first second.

The immediate posttransplant course was uneventful, and the patient was maintained on a triple immunosuppressive therapy consisting of tacrolimus, everolimus, and prednisolone. Four days after the surgery, she developed angina pectoris with elevated heart enzymes, but without any coronary lesions in the angiogram. Graft function was untainted during this incident, and the patient's pulmonary function parameters were satisfactory (Table 1 and Figures 1(a) and 1(b)).

**Figure 2:**
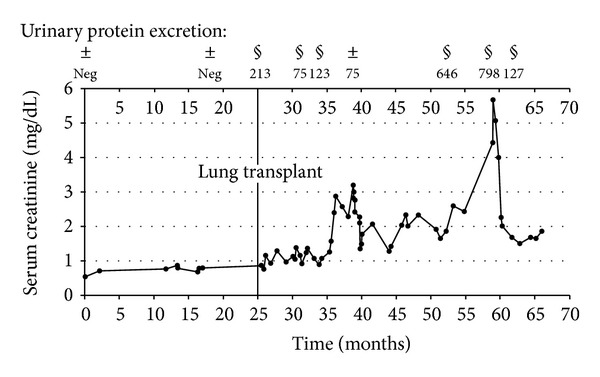
Time course of serum creatinine (mg/dL) and proteinuria, respectively. Urinary protein excretion measured by ^±^urinary dip stick (mg) and ^§^protein/creatinine ratio (mg/g).

However, during routine follow-up visits, worsening renal function became evident. This was, at that time, explained to be most likely due to a combination of the nephrotoxic effects of the immunosuppressants, the patient's diabetes mellitus, that became incident after transplantation, and her vascular disease. Notably, before her presentation in 2006, the renal function was normal, and there was no evidence of proteinuria or hematuria (Figure 2). 

One year after transplantation, the patient was admitted to the hospital with pneumonia of the native right lung and a doubling of the serum creatinine. She recovered with intravenous antibiotic, and the serum creatinine decreased to prior levels. Since the patient reported several episodes of diarrhea in the previous weeks, it was concluded that the rise of creatinine could be explained by dehydration too.

**Figure 3:**
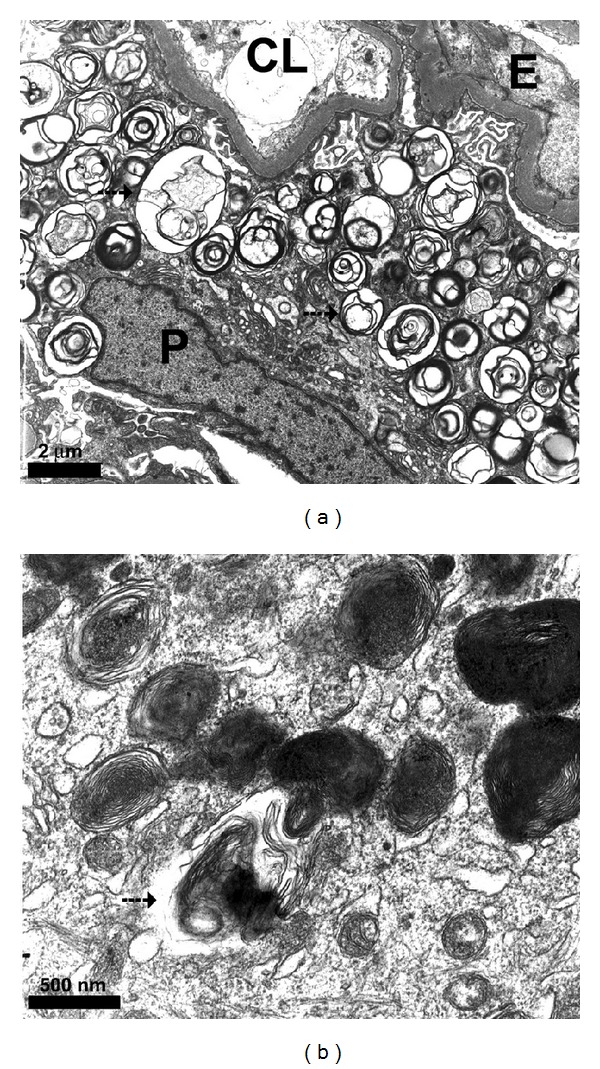
Electron microscopical changes in kidney (a) and native lung (b) biopsies. Glomerular podocytes (P) show extensive accumulation of “myeloid” and “zebroid” bodies (arrows) characteristics of Fabry diseases. However, capillary loops (CL) remain open, and endothelial cells (E) do not exhibit morphological changes. Ciliated pulmonary epithelial cells show an increase and accumulation of irregularly shaped inclusions in lysosomes and autophagosomes consistent with storage of sphingolipids (arrows).

Three years following transplantation, she developed a second episode of acute on chronic renal failure, again related to diarrhea. By that time, the serum creatinine was 5.68 mg/dL, the blood urea nitrogen level was 69.4 mg/dL, and mild proteinuria with a protein to creatinine ratio of 798 mg/g was present. Ultrasound showed normal-sized kidneys with a thinning of the parenchymal structure, appearing inhomogeneous and hyperechogenic. At the caudal pole of the left kidney, a cyst with a diameter of one centimeter was present. A renal biopsy revealed the definite histological diagnosis of Fabry disease of the kidney (Figure 3(a)). 

The diagnosis was confirmed biochemically with diminished enzymatic activity of *α*-galactosidase A in leukocytes (3 nmol/mg protein/hour; reference value: ≥51) and elevated urinary globotriaosylceramide excretion over 24 hours (1.07 nmol/mg lipid; reference value: 0–0.23). Consequently, genetic testing confirmed a *GLA* mutation at the nucleotide position 5234 (exon 2: g.5234G>A, c.335G>A, p.Arg112His) described in the literature [9]. 

In view of the confirmed diagnosis of a systemic lysosomal storage disorder, the explanted left lung and the remaining right lung were reexamined with regard to the presence of Fabry disease. Histology of the explanted left lung showed changes consistent with COPD. Retrospective electron microscopical analyses of “foamy” cells present in this lung specimen were inconclusive since only formalin-fixed, paraffin-embedded tissue was available for examination. Transfer of this material led to artifacts and loss of specific structures including “myeloid” or “zebroid” bodies characteristic in Fabry disease. Consequently, samplings of a total of 5 biopsy specimens from different areas were obtained by selective bronchoscopy of the native right lung to exclude sampling error since changes are considered focally accentuated. The biopsies were investigated by transmission electron microscopy only. Most areas did not exhibit specific pathological changes; however, in some ciliated cells, an accumulation of autophagic or “myeloid-like” inclusion bodies was seen (Figure 3(b)). Although this was not considered entirely diagnostic for Fabry disease, it was, in view of the patient's history, regarded as changes in keeping with the diagnosis.

An analysis of the index patient's history with regard to signs and symptoms revealed, at first sight, no evidence specific for Fabry disease. The patient experienced neither episodes of pain nor the postulated fever crisis. The quality of life was mostly impaired because of her advanced pulmonary disease. However, there had been several symptoms that, in retrospect, may be explained by her underlying disease. She reported dizziness for several years and had the impression of decreased appropriate sweating. So far she had not noticed any skin changes, but for several months, she felt tingling sensations in her lower legs. This was accompanied by muscle pain after a 15-minute time span of normal walking. At the age of 58 years, a cataract surgery was performed bilaterally, due to, as was hypothesized at the time, age-related degeneration of the lenses amplified by long-term steroid therapy. Notably, the patient had a history of angina pectoris and a suspected non-ST-elevation myocardial infarction that lacked coronary lesions detectable by angiography. This, however, fits, with hindsight, perfectly the symptoms of cardiomyopathy related to Fabry disease. Also, the recurrent diarrhea and abdominal pain may be explained by a globotriaosylceramide-related impairment of the intestinal tissue regularly seen in Fabry disease. However, gastrointestinal symptoms are also well-known side effects of immunosuppressive medication.

A recent physical examination showed a 62-year-old female with a body weight of 67 kg and a height of 156 cm (BMI: 27.5 kg/m^2^), presenting in an overweight constitution with stable vital signs. The average 24-hour blood pressure under antihypertensive medication was 135 over 78 mm mercury without decrease during night-time sleep. The neurological exam showed signs of neuropathy with an increase in the vibration perception threshold. On auscultation, a grade 2/6 systolic murmur was heard, and pulmonary auscultation revealed bilateral mild rales.

**Figure 4:**
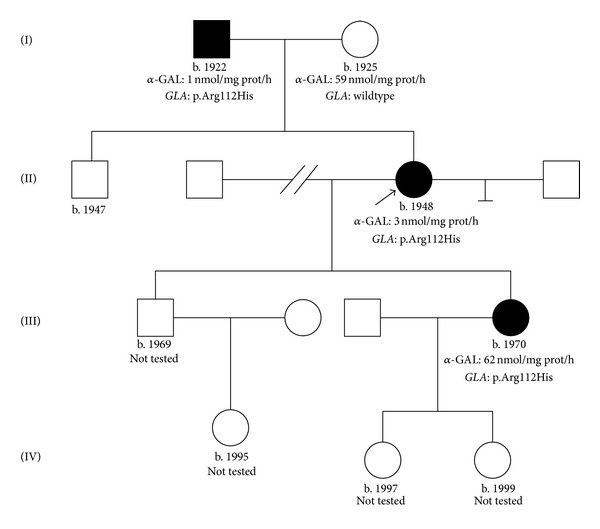
Pedigree. Latin letters indicate generations; ■ indicates affected males; □ indicates unaffected males; • indicates unaffected females; ∘ indicates unaffected females; ⊥ indicates no children; // indicates divorce; b.: year of birth; AGAL: *α*-galactosidase A activity; *GLA*: respective *GLA* mutation.

Recent electrocardiograms showed a normofrequent sinus rhythm with normal PQ and QRS intervals. However, repolarisation disturbance with various patterns was present. While exercise testing, the patient showed no signs of cardiac ischemia but a severe diminished physical performance due to dyspnoea. The heart rate was 91 per minute under beta-blocker therapy. Concentric left ventricular hypertrophy with normal left ventricular function was detected by echocardiography. Mild hypokinesia at the basal inferior septum and posterobasal wall was present. In computed tomography of the chest, the upper lobe of the transplanted lung appeared scarred but, in general, with no abnormality, particularly no signs of air trapping. In contrast, the native lung showed severe emphysema with mild peripheral consolidation, most likely of inflammatory origin. Nerve conduction velocity was decreased in upper and lower extremities. The MRI of the brain showed periventricular white matter lesions as well as T2-weighted alterations in the pons. Ultrasound of the carotid and vertebral arteries revealed no stenosis but slight unsteadiness of the endangium. The skin showed no evidence for angiokeratoma, and also no typical alterations of the patient's eyes could be detected aside from the cataract surgery. Notably, she suffered from frequent watery diarrhea and stomach pain with no morphological correlation to a 2010 performed rectocoloskopy. Only a slight rash in the cecum and sigma could be detected, which is why no biopsy was taken. The family history identified two further relatives with the respective mutation (father and daughter of the index patient) and additional four individuals with potential risk to carry the mutated variant of the *GLA* gene. The pedigree of the family is depicted in detail in Figure 4.

Among affected family members, her 40-year-old daughter has a long-standing clinical diagnosis of inflammatory bowel disease that appears to be related to the disordered sphingolipid metabolism. Although she reported burning pain in her hands and feet, neurological examination was normal. No abnormalities were found in the nerve conduction velocity or in the ultrasound of the carotid and vertebral arteries. In addition, MRI showed several suspect T2-weighted hyperintense lesions in the medullar layer. Of note, the otherwise healthy woman suffered a sudden hearing loss in 2008, which also fits to a diagnosis of Fabry disease. However, her serum creatinine was 0.86 mg/dL with physiological amounts of urinary protein excretion. Echocardiography showed no evidence of left ventricular hypertrophy, and pulmonary function was normal.

Interestingly, the 88-year-old father of the index patient, who worked as a carpenter until the age of 60 years, presented mainly asymptomatic, although his *α*-galactosidase A activity in leukocytes was only 1 nmol/mg protein/hour. In the year of 2010, the patient with a history of diabetes mellitus and arterial hypertension had a serum creatinine of 0.86 mg/dL and only mild proteinuria (338 mg/g creatinine). Echocardiography showed a mild concentric left ventricular hypertrophy, and the pulmonary function testing was largely normal. The mild course of the disease is consistent with previously described cases with the same mutation, usually referred to as attenuated phenotype [9].

## 3. Discussion

The case described here adds a new chapter to the often inexplicable clinical presentation of Fabry disease. We report the first patient with end-stage lung disease who received a lung transplant, unaware of her multiorgan involving metabolic disease. Similar startling cases about renal transplant donors who had a *GLA* mutation are reported in the literature [10, 11]. This clinical presentation in connection with the new established diagnosis of Fabry disease points to some important issues.

Pulmonary involvement in Fabry disease is often neglected and dyspnoea most commonly assigned to cardiac impairment. As such, lung disease is not much appreciated in the literature. However, results of the two registry databases (Fabry Outcome Survey (FOS) and Fabry Registry) report that 69% of male and 65% of female patients suffer from dyspnea [12] and 3% of male and 2% of female patients stated respiratory symptoms at their baseline evaluation [13]. 

In prospective studies, dyspnoea, cough, and wheezing are present in approximately one quarter of subjects, along with obstruction in pulmonary function testing (18%–38% [2–4]), which is predominantly unrelated to smoking. Interestingly, in particular in male patients with Fabry disease, all spirometric parameters worsened significantly with an increase in age compared to controls [4]. Of note, in 30%–63%, pulmonary function tests improved significantly after the application of bronchodilative agents [2, 4].

All studies except for one [4] found a decreased forced expiratory flow at 25% to 75% (FEF_(25–75)_), and that is in line with the presence of a small airway dysfunction in the majority of patients with Fabry disease. In the study performed by Magage et al., the decline of FEF_(25–75)_ (−8.3  ±  12%; median followup over 24 months) over time was the most prominent finding.

Histologically, globotriaosylceramide accumulation is mainly seen in bronchial smooth muscle cells, which in turn leads to hypertrophy [14]. However, also the epithelium of the airways including the ciliated cells shows significant amounts of lipid storage. Alveolar epithelial cells are spared, which fits the clinical picture of gas exchange impairment being more the exception than the rule [5, 15]. Less is known about globotriaosylceramide accumulation in capillaries, but other types of pulmonary vessels do not differ from storage patterns present in other tissues. Histological and electron microscopical examinations of our patient's renal biopsy showed changes characteristic of Fabry disease. In contrast, the evaluation of her native lung was less conclusive. However, ciliated airway epithelia exhibited increased numbers of “myeloid” bodies; these changes were not pronounced and only seen in two areas. However, characteristic changes may be by focally distributed and sampling error may be high, particularly in lung tissue.

Although pulmonary involvement seems to be regularly occurring in Fabry disease, physicians treating these patients focus predominantly on renal, cardiovascular, and cerebrovascular involvement. The reason that lung involvement has been widely neglected may be twofold. First, lung tissue seems to exhibit a better reserve capacity, and, secondly, decreasing lung function due to cardiac failure might in most cases be more likely. Prior to the introduction of sufficient renal replacement therapy, the leading cause of death was renal failure. Nowadays, the adverse outcome is determined by cardiac disease, primarily malignant arrhythmias and congestive heart failure [16]. 

Indeed, the case presented here is the first of lung failure due to severe COPD in a patient with Fabry disease. Since her right lung showed better residual function, she received a single left lung transplant as it is recommended for patients with COPD. Currently, both registry databases do not have any entries about lung-transplanted patients with Fabry disease (personal correspondence with Birgit Pareiss, a holder of the M.D. degree, Head of Business Unit Personalized Genetic Health & Endocinology, Genzyme GmbH, Austria, and Marie Allard, Global Project Manager FOS, Shire HGT, Sweden), and also the International Society of Heart and Lung Transplantation (ISHLT) Transplant Registry lists no documented case of a patient with lung transplant suffering from Fabry disease (personal correspondence with Leah B. Edwards, a holder of the Ph.D. degree, ISHLT Transplant Registry, Associate Director for Biostatistics).

This said, two cases with respiratory impairment requiring enzyme replacement therapy have been reported [17, 18], and in a small prospective randomized trial, a tendency to improvement in patients under enzyme replacement therapy could be observed, though the sample cohort was too small for a meaningful statistical analysis [15].

As becomes apparent in the case discussed here, the genotype-phenotype correlation in Fabry disease is highly variable, not only between unrelated subjects with the same mutation but also within families. In this case, the p.Arg112His genotype could be identified in the index patient and her father and daughter. While the male individual presented asymptomatic, both females complained about gastrointestinal and neurological symptoms. The index patient very likely had preset lung and kidney impairment due to the glycosphingolipid storage, which escalated by additional exogenous toxic exposure. The mutation is reputed to usually cause a mild phenotype, which in a way is confirmed by the remarkably high frequency (2 out of 34.736) of the mutation seen in a recently performed newborn screening [9, 19]. However, some individuals present with a more severe phenotype, and to some surprise, these are not necessarily males as demonstrated in the here-described family. The case reported by Ishii et al. [20] displays a severely affected 14-year-old boy harboring the respective missense mutation in addition to a functional polymorphism (p.Glu66Gln) frequently seen in the Asian population. 

In our center, further 6 families (19 patients) comprising the p.Arg112His genotype are known. Five of the 6 index patients were identified by kidney biopsy and one by means of a case-finding study in a cohort of renal transplant recipients. At least, 4 patients reached end-stage renal disease, underscoring the disease-causing impact of the mutation. Aspects like unknown modifier genes or undetected additional polymorphisms might play a crucial role, not to forget the even more heterogeneity produced by the random X-inactivation in heterozygous individuals. The patient described shows no major cardiac impairment and developed late onset kidney disease, although biopsy showed marked renal involvement with evidence of glomerular and tubulointerstitial scarring. This case's history demonstrates that the unfavorable effects of smoking set a fatal ball rolling leading to end-stage lung and kidney diseases. In light of the new evidence, it is very likely that both the progression to end-stage COPD and chronic renal failure were partly driven by Fabry disease. Also, concomitant illnesses like episodes of angina pectoris, cataract, and the history of recurrent diarrhea fit into the spectrum of possible manifestations of Fabry disease and could thus present symptoms of a single disorder.

## 4. Conclusion

We here report the first Fabry patient with end-stage lung disease who successfully received a lung transplant. Consequently, we identified at least two further affected relatives with a relatively mild phenotype, consistent with the p.Arg112His genotype.

Although the association of a lung transplant and Fabry disease appears far-fetched on first sight, respiratory impairment cannot be denied in Fabry disease. With this case presentation, we would like to stimulate discussion about rare differential diagnoses hidden beneath widespread diseases and that a correct diagnosis is the base of an optimal treatment strategy for each patient. Overall, the patient might have benefited from specific enzyme replacement therapy, especially in view of the chronic kidney disease.

## Figures and Tables

**Table 1:** Details of pulmonary function testing between 2005 and 2010 (numbers are given in percent of the predicted value).

	Jul.	Feb.	Sep.	Sep.	Sep.	Sep.
	2005	2007	2007	2008	2009	2010
FVC	25.8	41	39	40.5	50.3	41.6
FEV_1_	12.8	13	45.1	43.8	52	44.9
MEF_75%_	10.2	n.d.	47.4	45.8	47.1	48.1
MEF_50%_	4.8	3	62.9	44.4	44.6	48.4
MEF_25%_	4.4	n.d.	81.5	41.2	41.4	38

FVC: forced vital capacity; FEV_1_: forced expiratory volume in first second; MEF_75%_: maximum expiratory flow at 75%; MEF_50%_: maximum expiratory flow at 50%; MEF_25%_: maximum expiratory flow at 25%.

“n.d.” indicates “not done.”
